# Illinois Charitable Eye and Ear Infirmary

**Published:** 1877-02

**Authors:** 


### Clinical Reports.

### ILLINOIS CHARITABLE EYE AND EAR INFIRMARY.

### SERVICE OF DR. F. C. HOTZ.

1. *A Piece of Iron in the Iris 24 years.*

In July of last year a Mrs. M. Sch., æt. 29, presented herself at the infirmary, because, for the past four weeks, her left eye had been getting red and sensitive. By ordinary daylight we noticed a slight amount of redness around the cornea; the latter appeared perfectly clear, the pupil was round and movable, and the lens exhibited all signs of a mature soft cataract. By the aid of the focal illumination, however, we discovered, in the external half of the iris, a small dark brown fragment of iron with sharp corners and edges; it was lodged midway between the periphery and the pupillary border of the iris, and had evidently pierced the entire thickness of the latter, so as to wound the anterior capsule of the crystalline lens. For, after dilating the pupil by atropia, we found that portion of the capsule which was behind the injured portion of the iris to be drawn out like a funnel toward, and fastened to, the iris. But the pupillary border of the iris was perfectly free, there being no posterior synechiæ. The focal illumination also revealed the point where the iron had passed through the cornea; it was indicated by a fine linear scar just opposite the foreign body in the iris.

At first the woman could not tell how she lost the sight of this eye, she only knew it had never been sore until lately; but after giving the question, as to the possibility of an injury a few minutes thought, she remembered that when she was about four or five years old a small fragment of a hatchet flew into her eye, and an old woman had tried to take it out. She always believed it had been removed because she felt no pain in the eye afterwards, and because the eye never was inflamed.

The absence of iritis in this case is a remarkable fact, because, as a rule, the iris does not tolerate the presence of a foreign body; sooner or later this excites an inflammation of the iris which can be arrested only by the removal of the foreign substance. And the final history of this case shows that it is no exception of this rule; although the eye had tolerated the fragment of iron during a period of 24 years, it then began to show the signs of iritic irritation, which was likely to lead to a destructive inflammation unless the foreign body was removed in time.

The patient has not returned after her first visit; and it might be possible that the use of atropia had temporarily checked the irritation; may be, however, that she dreaded too much the idea of an operation.

2. *A Splinter of Coal in the Posterior Layers of the Cornea.*

On October 14th, an Irish boy, æt. three years, was brought to the infirmary. Three days ago another boy had thrown a small piece of coal at him; the coal hit his right eye and broke into several pieces; one piece, at least, was taken out of the eye by the boy's father. But when the eye, instead of getting well, became redder, more painful, and very sensitive to the light, the parents sought for advice at the infirmary.

The eye exhibited the usual symptoms of corneal inflammation, which was occasioned by a small wound in the inner half of the cornea. This wound was made by a very small splinter of coal, which had cut its way obliquely backward through the corneal tissues until it reached Descemet's membrane. There it was arrested, and there it lay with its sharp edge grating on that very delicate membrane; it could gradually preforate it and fall into the anterior chamber. The slightest touch with an instrument also would have pushed it through the posterior layer of the cornea into the aqueous humor.

In order to prevent this untoward accident we proceeded in the following manner: The boy being etherized the wound was enlarged by cautious strokes with a cataract knife, so as to change its oblique and narrow track into a short and wide funnel, its narrow end being turned toward the foreign body. The wound thus being made larger than the largest diameter of the foreign body, the latter could easily escape if moved by a slight pressure from behind. This pressure could not be applied by an instrument, inasmuch as any attempt to insert it between the splinter and Descemet's membrane would necessarily have ruptured the latter and have dislodged the coal into the anterior chamber. But we could utilize the pressure of the aqueous humor for the motor power. For this purpose the Descemet's membrance was punctured a little to one side of the foreign body, and the puncture was enlarged by carrying the point of the knife (which was then in the aqueous humor) toward and behind the coal. By a gentle pressure upon the lower border of the wound with the blade of the knife the puncture gaped sufficiently to permit the escape of the aqueous humor. This current of water, directed toward the posterior surface of the splinter was strong enough to dislodge the latter and to carry it up toward the anterior surface of the cornea. As soon as it had fairly receded from the posterior surface of the cornea the pressure upon the wound with the knife was discontinued, in order to stop the further escape of the aqueous humor. The splinter was now near the anterior surface of the cornea, and could be easily and safely removed by the point of the knife while it was withdrawn from the anterior chamber. It was a minute thin particle of hard coal.

By arresting the flow of the aqueous humor before the anterior chamber was entirely emptied, we prevented the anterior surface of the iris from coming in direct contact with the posterior surface of the cornea, and obviated thereby the possibility of an adhesion of the iris to the cornea at the point where it had been punctured. The operation over (which did not occupy a quarter of the time it takes to describe it) a drop of a solution of atropia was instilled, and the eye carefully bandaged. Three days later all signs of inflammation had disappeard, the corneal wound was closed by a fine scar, the anterior chamber of a normal depth and the pupil regular and still dilated by the effect of atropia.

3. *Paralysis of Ocular Muscles after Diphtheria.*

During the present epidemic of diphtheria it may be very appropriate to draw the attention of the physicians to the fact that a paralysis of the muscles of the eye is occasionally met with after diphtheria. It may follow a mild or severe attack of the disease and it may settle upon any of the external muscles, or affect principally the ciliary muscle. Under a general treatment with tonics the function of the affected muscle will, as a rule, be restored.

The following cases have lately come under our observation:

*a. Paralysis of the Right External Rectus Muscle.*

Fritz R., æt. four years, was seized with a violent attack of diphtheria in November; he was sick almost four weeks. When he was convalescent his parents noticed that his left eye was turned inward. Examined December 23d, a marked convergent squint of the left eye, due to a partial paralysis of the external rectus muscle. Ordered the elixir of calisaya bark and iron.

January 27th. The eyes are straight to all appearance, and by covering the left eye with the hand, so as to exclude it from vision, no deviation can discovered.

*b. Paralysis of the Ciliary Muscle.*

Gustav N., æt. nine years, had a mild attack of diphtheria in the latter part of November. After recovery he complained of being unable to read ordinary print. The parents unable to notice anything abnormal in the eyes, suspected the boy of malingering in order to be excused from school.

Examination, Jan. 1st. Pupils rather large and decidedly sluggish in responding to the change of light. No inflammation, no obscuration of the transparent media, normal fundus. $V=\frac{20}{20}$ with convex 24; yet with these glasses he can read Jaeg. 12 only, but with convex 8 he is able to read Jaeg. 1 fluently at a distance of 12 inches.

The paralysis of the ciliary muscle obliterated the power of accomodation; the eyes being naturally far-sighted, required the aid of a convex lens (+24) to adjust the focus at distant vision (at 20 feet). And to adjust the focus for the short distance of 12 inches an *additional* lens (+12) was required; *i. e.* $\frac{1}{24}+\frac{1}{12}=\frac{1}{8}$.

*c. Paralysis of the Ciliary Muscle.*

Stella N., æt. seven years, Gustav's sister, also had diphtheria at the same time, and suffered from weakness of the accommodation (partial paralysis of the ciliary muscle).

*d. Partial Paralysis of the External Rectus.*

Another sister, æt. five years, had a marked convergent squint of the left eye after diphtheria. At the time she was examined the external rectus muscle had recovered its power to a great extent, showing a deficiency only under the covering hand.

